# Prevalence of Three-Rooted Mandibular First Molars among Indians Using SCT

**DOI:** 10.1155/2013/183869

**Published:** 2013-06-11

**Authors:** Amit Kumar Garg, Rajendra Kumar Tewari, Neha Agrawal

**Affiliations:** ^1^Department of Conservative Dentistry and Endodontics, Modern Dental College & Research Centre, Indore, India; ^2^Department of Conservative Dentistry and Endodontics, Dr. Z. A. Dental College, AMU, Aligarh, India; ^3^Department of Public Health Dentistry, Modern Dental College & Research Centre, Indore, India

## Abstract

Undetected extra roots or root canals are a major reason for failure of endodontic treatment. Failure to recognize an extra distolingual (DL) root in mandibular first molar may lead to incomplete debridement of the root canal system and eventually treatment failure. Therefore, it is crucial that atypical anatomy is identified before and during dental treatment. Spiral computed tomography (SCT) images can show 3D images, and therefore much detail can be used when traditional methods prevent adequate endodontic treatment. The overall incidence of DL roots on the mandibular first molars was 6.40% for all patients and 5.00% for all teeth, respectively. The occurrence of DL roots on the right side and on the left side showed a statistically significant difference. The bilateral incidence of symmetrical distribution of DL roots was 56.25%. The DL root canal orifice was separated from DB canal orifice by 2.79 ± 0.34 mm, from the MB canal orifice by 4.23 ± 0.81 mm, and from the ML canal orifice by 3.29 ± 0.52 mm. The high prevalence of the DL root in permanent mandibular first molars among the Indian population by using SCT and estimations of the interorifice distance of such teeth might be useful for successful endodontic treatments.

## 1. Introduction

The treatment of the entire root canal system is essential to maximize the possibility of success in endodontic therapy. It is necessary for the clinician to have a thorough knowledge of the dental anatomy as well as of its variations. Undetected extra roots or root canals are a major reason for failure of root canal treatment [1]. Therefore, it is imperative that the aberrant anatomy is identified for successful root canal treatment of mandibular molars.

Anatomical variations are an acknowledged characteristic of mandibular permanent molars. Mandibular first molars typically have two roots placed mesiodistally, but they sometimes have an additional distolingual (DL) root a radix entomolaris (RE), usually on the distolingual aspect [2, 3]. An RE can be found in the first, second and third mandibular molars, occurring least frequently in the second molar [4]. This extra root is typically smaller than the distobuccal root and is usually curved, requiring special attention when root canal treatment is being considered for such a tooth [2–4]. 

De Moor et al. (2004) classified the RE into three types according to the buccolingual variations [2]. Type I refers to a straight root, Type II to an initially curved entrance that continues as a straight root, and Type III to an initial curve in the coronal third of the root canal, followed by a second curve beginning in the middle and continuing to the apical third. Recently, Song et al. (2010) classified the RE into 5 types according to their morphological characteristics [4]. Two additional types are Type IV (small type), where root length less than half that of the distobuccal root, and Type V (conical type), which is a cone-shaped extension with no root canal.

An extra DL root in the mandibular first molar is associated with certain ethnic groups. In White population, an extra DL root is present in mandibular first molars with a frequency of 3.4%–4.2% [5–8]. Recently, Schäfer et al. (2009) found that an extra DL root occurs in 1.35% of German patients [9]. In Black population, it occurs in 3% of cases [10]. In Eurasian and Indian population, Tratman [11] found that it was less than 5% while in recent study [11] Garg et al. (2010) found that it was 5.97% among the Indian population [12]. In Populations with Mongoloid traits such as Chinese, Taiwanese, Eskimo, Korean, and American Indians, DL roots occur with a frequency of 5%–40% [4, 13–19]. This indicates a high ethnic variation among different groups. The RE is considered to be a normal morphologic variant or eumorphic root morphology due to its high frequency in Asian populations [3] and can be seen as a predominantly Asiatic trait [16]. Among Whites, RE is not very common [20] and is considered to be unusual or dysmorphic root morphology.

The purpose of this retrospective study was to determine the incidence of DL roots in mandibular first molars, their gender and side-related differences, and evaluate their morphology among the Indian population, identified by SCT.

## 2. Material and Methods

SCT images from 348 subjects that had previously been obtained in the Department of Radio-Diagnosis (CT/MRI Centre), J.N. Medical College, Aligarh Muslim University, Aligarh, India, during the period of December 2008-June 2011 were screened and examined. SCT images of 500 mandibular first molars in 250 patients (130 female and 120 male) possessing bilateral mandibular first molars were taken. The CT images were obtained using SCT scanner (Somatom Balance, Siemens, Erlangen, Germany). The exposure settings were between 130 kV and 90–135 mA with an image resolution of 1012 × 688 pixels. Three-dimensional images displayed on a LCD monitor were inspected by 2 endodontists. All images of permanent mandibular first molars were investigated by moving the toolbar from the crown down to the apex in an axial direction to explore the number of roots [19]. Disagreement in the interpretation of images was discussed between the 2 endodontists until a consensus was reached [18].

The age and the sex of each patient were recorded. In teeth with three roots, the position of the third root was recorded. The criteria for subject selection were the following: (1) each subject had to have fully erupted permanent mandibular first molars bilaterally; (2) the permanent mandibular first molars had to have fully formed apexes, no root canal fillings, posts, or crown restorations. The relative incidence of DL roots on mandibular first molars in the patients; their correlations between the right-side and the left-side occurrences and between female and male patients were analyzed by using the *z*-test. The bilateral incidence of DL roots on mandibular first molars was also enumerated.

The 4 canal orifices were marked as spots; then the distance values between 2 orifices are measured automatically in millimeter by the software (Syngo fastView). The distances from the DL canal orifice to the DB, MB, and ML canal orifices of the 25 permanent three-rooted mandibular molars were measured and collected, with the obtained values compared by using the *t*-test.

## 3. Results

**Figure 1:**
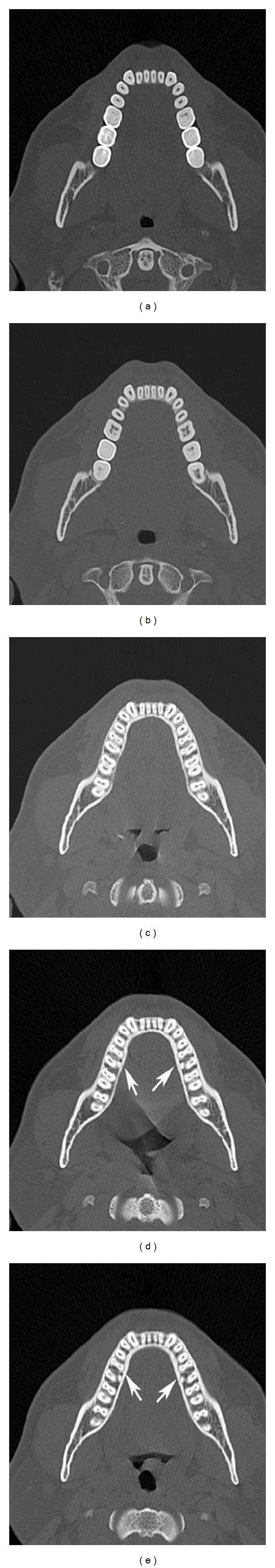
Axial CT scans of bilateral permanent three-rooted mandibular first molars. (a) Coronal section, (b) pulp-chamber section, (c) coronal-third root section, (d) mid-root section, and (e) apical-third root section. Arrows indicate bilateral extra DL roots.

**Figure 2:**
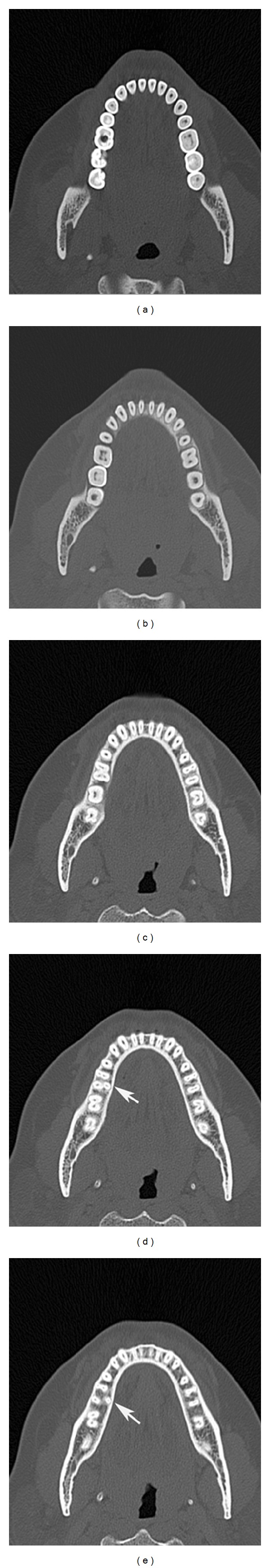
Axial CT scans of unilateral permanent three-rooted mandibular first molar. (a) Coronal section, (b) pulp-chamber section, (c) coronal-third root section, (d) mid-root section, and (e) apical-third root section. Arrows indicate extra DL roots.

A total of 250 patients (130 female and 120 male) aged between 12 and 65 years (mean age of 30.75 ± 14.41 years) were evaluated in this study. A total of 500 mandibular first molars were evaluated and the results were tabulated (Table 1). Gender-related difference (*z* = 0.71, *P* > 0.05) was not found to be statistically significant for the incidence of DL roots on mandibular first molars. The incidence of extra DL roots on the mandibular first molars was 6.92% for female patients and 5.83% for male patients. The overall incidence of DL roots on the mandibular first molars was 6.40% for all patients and 5.00% for all teeth, respectively. The left mandibular first molar in male patients had the lowest incidence of an extra DL root (0.0%); the bilateral occurrence of DL roots on mandibular first molars in female patients had the highest incidence (3.85%). The occurrence of DL roots on the right side (2.40%) and on the left side (0.40%) showed a statistically significant difference (*z* = 2.67, *P* < 0.05). The bilateral incidence of symmetrical distribution of DL roots was 56.25% (Figure 1). Of the 7 unilaterally occurring teeth, 6 teeth (37.25%) occurred on the right side and one tooth (6.25%) on the left side (Figure 2).

The DL root canal orifice was separated from DB canal orifice by 2.79 ± 0.34 mm (mean ± standard deviation), from the MB canal orifice by 4.23 ± 0.81 mm, and from the ML canal orifice by 3.29 ± 0.52 mm (Table 2). These distances did not differ significantly between the right and left mandibular first molars.

## 4. Discussion

In the present study, the prevalence of DL roots on mandibular first molars among the Indian population was investigated by using SCT. It was found that 6.40% of all patients and 5.00% of all teeth examined, respectively, had an extra DL root (Table 1). This percentage is higher than those found by previous study of Indian subjects by using periapical radiographs [12]. Somogyl-Csizmazia and Simons (1971) found a higher figure 15.6% among Canadian Indians [21]. Tratman [11] and Turner (1971) found a lower figure (0.20%) among Asiatic Indians [11] and (5.8%) among American Indians [22], respectively.

In the present study, gender was not found to be a statistically significant factor (*P* > 0.05), which is similar to results of recent studies [9, 12, 17, 18]. In many previous studies, DL roots of first molars were described to have a predilection toward the right side [4, 5, 11, 15, 17–19, 23, 24], whereas a left side predilection has been reported much less frequently [25]. In this present study, DL root was found more frequently on the right side by using SCT images, whereas in some studies [9, 12] there was no side-related significant difference by using periapical radiographs (right versus left side, *P* > 0.05). The incidence of bilateral DL roots of mandibular first molars was 56.25%. This value is close to reported figures of 50.00%–68.57% of much research on Asian population [4–6, 15–19, 23] and more than the recent study by Garg et al. (2010) among Asiatic Indians (37.14%) by using periapical radiographs [12]. These contradictory findings may be explained by marked differences in the sample size and in the methods used, making further investigations necessary.

In previous studies evaluating the prevalence of DL roots, mainly two different methods have been used. Several researchers have studied this directly from extracted teeth [11, 13, 20, 24–28] while others have used a radiographic technique [9, 12, 15, 17, 21, 29, 30]. In study of extracted teeth, it is impossible to compare results relating to gender and side-related differences for DL roots; the latter method using IOPA radiographs is noninvasive and allows for comparisons relating to gender and side-related differences. According to Walker and Quackenbush (1985), normally a third root should readily be evident in about 90% of cases radiographically, but occasionally it might be difficult to see because of its slender dimensions [15]. 

Conventional radiographs give only a 2-dimensional view of the teeth, whereas spiral computed tomography (SCT) can show detailed 3-dimensional images for use in endodontic applications and morphologic analyses. Recently, Jayasinghe and Li 2007, Tu et al. (2007) and Song et al. (2010) investigated the incidence of DL roots using CT scan [4, 18, 19]. SCT images seem to be more conservative, accurate and look to be a promising tool for investigating the prevalence and morphological features of DL roots in molars. In this study, the CT images gave a detailed and accurate picture of molar teeth, which was likely to have been more reliable than the other methods, but the major concern is the radiation dosage. The radiation dose of intraoral periapical radiograph is considerably lower (0.005 mSv) than mandibular CT scan (1.32 mSv) [31]. Recently, CBCT has become available and is characterized by the rapid acquisition of volume images from a single low radiation dose (0.034–0.102 mSv) scan of the patient, in contrast to SCT [19]. For comparison, the average annual effective dose from background radiation in the United States is about 3.0 mSv [32]. The disadvantage of CT over conventional radiographs is radiation exposure, so it is essential that radiation exposure is kept as low as reasonably achievable [33]. Patient positioning modifications (tilting the chin) and the use of additional personal protection (thyroid collar) can substantially reduce the dosage by up to 40% [34].

Dentists could use CT scan to obtain the 3D images of a tooth before or during root canal treatment for teeth with an extra DL root. Preparing appropriate straight-line access and locating the orifice of the extra DL root canal (located a mean of 2.79 mm from the DB canal orifice, 4.23 mm from the MB canal orifice, and 3.29 mm from the ML canal orifice in the present study) typically need modifying the classic triangular opening to a trapezoidal form to improve the localization of and access to the root canals. 

Based on the recent study [12], the prevalence of DL roots was 5.97% among the Asiatic Indians, which was much greater than 0.20% among Asiatic Indian [11]. The present study indicates a higher prevalence of DL roots (6.40%), with more than 50% of Asiatic Indian population exhibiting bilateral symmetrical occurrence of DL roots on mandibular first molars by using SCT. This information is critically important for specialized dental procedures such as root canal treatment, periodontal treatment, orthodontic movements and surgical extraction. 

## 5. Conclusion

Clinicians should be aware of high prevalence and bilateral symmetrical occurrence of the DL roots in mandibular first molars among the Indian population before and during the dental procedures. The mean interorifice distances might help endodontists to locate orifices and modifying access preparations. This might increase the success rate of dental treatment.

## Figures and Tables

**Table 1:** Numbers and percentages of patients with mandibular first molars having an extra distolingual root by gender, unilateral and bilateral status, and total occurrence.

	Unilateral				Bilateral		Total	
Number of patients	Right		Left					
	No.	%	No.	%	No.	%	No.	%
Female (130)	3	2.31	1	0.77	5	3.85	9	6.92
Male (120)	3	2.50	0	0.00	4	3.33	7	5.83
Total patients (250)	6	2.40	1	0.40	9	3.60	16	6.40
No. of total teeth examined (500)	6	1.20	1	0.20	18	3.60	25	5.00

**Table 2:** Distances from DL orifice to other 3 orifices in 25 permanent three-rooted right and left mandibular first molars.

Teeth	Distance (mm)		
	DLO-DBO	DLO-MBO	DLO-MLO
# 36 (*n* = 10)	2.80 ± 0.41	4.18 ± 0.79	3.25 ± 0.52
# 46 (*n* = 15)	2.79 ± 0.27	4.28 ± 0.83	3.33 ± 0.53

Total (*n* = 25)	2.79 ± 0.34	4.23 ± 0.81	3.29 ± 0.52

Distolingual orifice (DLO); distobuccal orifice (DBO); mesiobuccal orifice (MBO); mesiolingual orifice (MLO).
